# Inflammation and Atherosclerosis

**DOI:** 10.3390/cells10051197

**Published:** 2021-05-14

**Authors:** Klaus Ley

**Affiliations:** 1 Laboratory of Inflammation Biology, La Jolla Institute for Immunology, 9420 Athena Circle Drive, La Jolla, San Diego, CA 92037, USA; klaus@lji.org; 2 Department of Bioengineering, University of California, 9500 Gilman Drive, MC0412, La Jolla, San Diego, CA 92093, USA

This 11-chapter Special Issue of *Cells* spans the gamut from basic science in mechanistic animal models to translational science to outcomes of clinical trials, all focused on the role of inflammation in atherosclerosis.

The basic science reviews cover inflammatory cytokines, chemokines and immune cell subsets. The cytokine chapter “Act Locally, Act Globally—Microbiota, Barriers, and Cytokines in Atherosclerosis” by Natalia Kurilenko, Aliia R. Fatkhullina, Aleksandra Mazitova and Ekaterina K. Koltsova starts by discussing the IL-1 family, including IL-1α, IL-1β, IL-18, IL-33, the IL-36 sub-family and IL-37 [1]. Of similar complexity, the IL-17 family includes IL-17A, IL-17B, IL-17C, IL-17D, IL-17E (also known as IL-25), and IL-17F. Almost all of these cytokines have been shown to play a role in atherosclerosis, usually pro-inflammatory. The IL-10 family, consisting of IL-10, IL-19, IL-20, IL-22, IL-24, IL-26, IL-28A, IL-28B, and IL-29, has diverse but mostly inflammation-dampening effects. IL-23 and IL-22 affect the species composition of intestinal microbiota, which, in turn, modulate atherosclerosis. The chemokine chapter “Inflammatory Chemokines in Atherosclerosis” is authored by Selin Gencer, Bryce R. Evans, Emiel P.C. van der Vorst, Yvonne Döring and Christian Weber, one of the leading groups in this field [2]. Among the panoply of chemokines, CCL2, 3, 5, CXCL1 and 4, CX3CL1 and the atypical chemokine MIF are the most important players, binding to overlapping sets of receptors. Cell-type specific knockouts have shed light on the specific functions of many of these receptors in atherosclerosis.

The cell types covered by the basic science reviews include B cells, macrophages, monocytes, CD4 and CD8 T cells. The review “Functional Role of B Cells in Atherosclerosis” by Shelby D. Ma, Marion Mussbacher and Elena V. Galkina shows that B cells have both pro- and anti-atherogenic functions, with isotype-switched B2 cells exacerbating atherosclerosis and IgM-secreting B1 cells being protective [3]. One specialized B cell subset is called innate response activator (IRA) B cells, a GM-CSF-secreting B cell type that promotes atherosclerosis. Other B cell subsets have regulatory, anti-inflammatory functions by secreting IL-10. “Monocyte Recruitment, Specification, and Function in Atherosclerosis” is covered by Ki-Wook Kim, Stoyan Ivanov and Jesse W. Williams, an emerging leader in this field [4]. The authors review the hematopoietic pathways that lead to monocytes and dendritic cells. Many macrophages, including those in the healthy artery wall, are not monocyte-derived but seeded before or shortly after birth. They self-renew by local proliferation. In atherosclerosis, monocyte-derived macrophages join the fray and endow the atherosclerotic vessel wall with multiple subsets or macrophages. The CD8 T cell review by Sarah Schäfer and Alma Zernecke is entitled “CD8+ T Cells in Atherosclerosis”, one of the more controversial areas of current atherosclerosis research [5]. CD8 T cells in the atherosclerotic artery wall can be activated, cytotoxic, or dysfunctional and exhausted. Cytotoxic CD8 T cells kill target cells by Fas-Fas ligand interactions and by cytotoxic granules. Removing CD8 T cells by genetic means or antibodies is generally atheroprotective. However, some CD8 T cells express CD25 and can have regulatory functions, which may explain why some groups reported opposite results, that CD8 T cells reduce atherosclerosis. The review provides a balanced view of the results from different groups using different mouse models. Two reviews are focused on CD4 T cells, one dealing with “Regulatory T Cell Stability and Plasticity in Atherosclerosis” by Amal J. Ali, Jeffrey Makings and Klaus Ley, addressing one of the key challenges to translating vaccination approaches: regulatory T cells (Tregs), whether natural or induced by vaccination, are not stable over time in an atherogenic environment [6]. Treg plasticity entails the acquisition of lineage-defining transcription factors of other CD4 T cell lineages. Treg instability refers to the eventual loss of the Treg-defining transcription factor FoxP3. Timoteo Marchini, Sophie Hansen and Dennis Wolf wrote a very up-to-date review on “ApoB-Specific CD4+ T Cells in Mouse and Human Atherosclerosis”, an emerging area of research that also holds promise for translation [7]. They discuss the CD4 T cell lineages (Th1, 2, 9, 17 and 22, Tregs, follicular helper T cells, cytotoxic CD4 T cells and CD4+ NKT cells). The evidence for autoantigen-specific CD4 T cell responses in atherosclerosis comes from MHC-II tetramers and from restimulation assays.

These two CD4 T cell reviews are nice lead-ins for translational reviews. One is entitled “Regulatory T Cell-Enhancing Therapies to Treat Atherosclerosis” by Hafid Ait-Oufella, Jean-Rémi Lavillegrand and Alain Tedgui [8]. This group discovered the role of Tregs in atherosclerosis in a landmark paper in 2006. In addition to classical CD4+ CD25+ FoxP3+ Tregs, they discuss IL-10-secreting type 1 regulatory T cells (Tr1), TGF-β-secreting type 3 Th cells (Th3), and CD8+ Tregs. The translational part of this review covers the most recent efforts aimed at stabilizing regulatory T cells in patients and model systems. A related translational review, “Vaccination in Atherosclerosis” by Felix Sebastian Nettersheim, Lauren De Vore and Holger Winkels explores the possibility of harnessing the adaptive immune system to prevent and perhaps reverse atherosclerosis [9]. Vaccines that would inhibit the LDL receptor-lowering protein PCSK9 work by inducing neutralizing antibodies. Another class of vaccines is aimed at inducing a regulatory T cell response to ApoB or other components of LDL. Currently, ongoing trials test whether low-dose IL-2 administration can stabilize regulatory T cells. The third translational review “Inflammation-Related Risk Loci in Genome-Wide Association Studies of Coronary Artery Disease” by Carina Mauersberger, Heribert Schunkert and Hendrik B. Sager summarizes many recent genome-wide association studies revealing the association of single nucleotide polymorphisms in well-known and surprising inflammatory molecules and pathways [10]. Nineteen loci in inflammation genes are now firmly established as atherosclerosis-modifying, including SNPs in genes for adhesion molecules, chemokine receptors and cytokines such as IL-6. Other SNPs significantly associated with atherosclerosis are in genes for receptors of the innate immune system. This genetic knowledge can inform therapeutic and preventative strategies in precision medicine approaches.

This Special Issue is capped by a clinical review on “Targeting Inflammatory Pathways in Cardiovascular Disease: The Inflammasome, Interleukin-1, Interleukin-6 and Beyond” by Peter Libby, the undisputed pioneer and leader in this field [11]. Starting with a history of IL-1 research, the inflammasome machinery and its regulation, and downstream effectors such as IL-6 and the acute phase C-reactive protein (CRP), Libby focuses on the successful CANTOS trial that showed a reduction in major adverse cardiovascular events in people with high C-reactive protein treated with Canakinumab, a monoclonal antibody blocking IL-1β function. This was the first successful trial of any anti-inflammatory therapy in patients with atherosclerosis. The COLCOT and LoDoCo2 clinical trials showed that low dose colchicine also had beneficial effects.

I wish to thank all authors for their thoughtful contributions and hard work. I am honored that I was entrusted with being the editor of this Special Issue and hope that basic, translational and clinical researchers will find it a useful and up-to-date reference.
